# Influence of the viscosity of healthy and diseased human mucins on the motility of *Helicobacter pylori*

**DOI:** 10.1038/s41598-018-27732-3

**Published:** 2018-06-26

**Authors:** Clover Su, Médea Padra, Maira Alves Constantino, Sinan Sharba, Anders Thorell, Sara K. Lindén, Rama Bansil

**Affiliations:** 1 Boston University, Materials Science and Engineering Division, Boston, 02446 USA; 2 University of Gothenburg, Department of Medical Biochemistry and Cell Biology, Göteborg, 41390 Sweden; 3 Boston University, Physics Department, Boston, 02215 USA; 4 Karolinska Institutet, Department for Clinical Science and Department of Surgery, Ersta Hospital, Stockholm, Sweden

## Abstract

We present particle tracking microrheology results on human mucins, isolated from normal surface and gland mucosa and one tumor sample, and examine the motility of *Helicobacter pylori* in these mucins. At 1.5% concentration human mucin solutions are purely viscous, with viscosity η (gland mucin) > η (surface mucin) > η (tumor mucin). In the presence of motile *H*. *pylori* bacteria, particle diffusion is enhanced, with diffusivity D_+bac_(tumor mucin) > D_+bac_(gland mucin) > D_+bac_(surface mucin). The surface and tumor mucin solutions exhibit an elastic response in the presence of bacteria. Taken together these results imply that particle diffusion and active swimming are coupled and impact the rheology of mucin solutions. Both J99 wild type (WT) and its isogenic ΔbabA/ΔsabA mutant swam well in broth or PGM solutions. However, the human mucins affected their motility differently, rendering them immotile in certain instances. The distribution of swimming speeds in human mucin solutions was broader with a large fraction of fast swimmers compared to PGM and broth. The bacteria swam fastest in the tumor mucin solution correlating with it having the lowest viscosity of all mucin solutions. Overall, these results suggest that mucins from different tissue locations and disease status differ in their microrheological properties and their effect on *H*. *pylori* motility.

## Introduction

**Figure 1:**
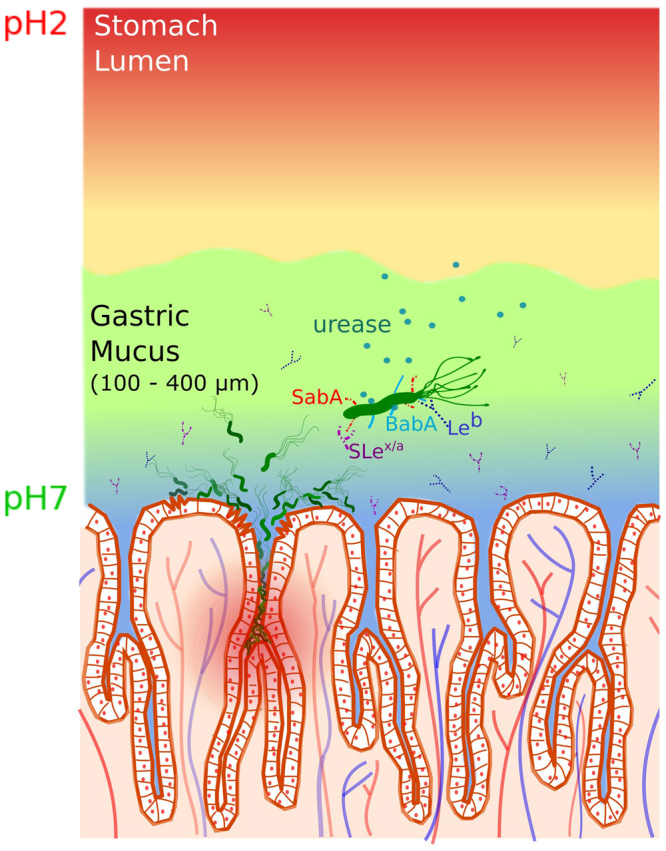
A schematic illustration of the gastric mucosa depicting the interaction of *H*. *pylori* with mucin. The cells on the mucosal epithelial surface are shown demarcating the crypts and the underlying vasculature. The pH gradient from 7 at the cell surface to about 2 in the lumen is indicated by the blue to red shading. A single bacterium is greatly enlarged to display the adhesins, BabA (blue) and SabA (red) which bind to specific antigens Le^b^ (blue symbol) and SLe^x/a^ (purple symbol) on the mucin, respectively. The urease (green circles) secreted by the bacterium enables it to hydrolyze acid, de-gel the mucus and swim across (see text). Bacteria colonizing on the cell surface as well as deep in the crypts are also shown.

*Helicobacter pylori* is a pathogen uniquely evolved to adapt to the harsh, highly acidic environment of the human stomach and the only bacteria known to cause cancer. While half of the world’s population harbors *H*. *pylori*, only 15–20% of them show symptoms of gastritis, gastric ulcers and cancer. To understand this observation, it is important to examine the key factors by which *H*. *pylori* survive and move through the acidic mucus layer to colonize the epithelium, interacting with the mucosa and the host immune system. Figure 1 shows an illustration of *H*. *pylori* in the mucosal layer with some of these factors.

Bacterial infection depends on the complex interplay between the physical properties of the gastric mucus layer and the biochemical interactions of the bacterium with mucin and other virulence factors such as cytotoxins which further trigger a cascade of immune responses from the host, lead to cell damage, inflammation or cancer. (See for example, Salama *et al*.^1^).

The mucus layer in the mammalian stomach has been shown to consist of two layers with distinct rheological properties^2^. The layer adherent to the epithelial cell surface, 100–150 µm in thickness, has higher mucin concentration and greater viscoelasticity, while the layer close to the lumen, 100–700 µm thick, has lower mucin concentration and is less viscous. Mucus in both layers is composed of MUC5AC mucin produced by the surface epithelium and MUC6 mucin produced by the glands^3–5^. In addition to colonizing on the surface epithelium, *H*. *pylori* is also found deep in gastric glands^6,7^ where the mucus is predominantly composed of MUC6. To get across the acidic environment in the vicinity of the gastric lumen and swim through the highly visco-elastic mucus layer, *H*. *pylori* secretes urease to hydrolyze urea^8,9^, which elevates the pH and helps the non-acidophilic bacterium to survive in the stomach^10^. Contrary to earlier suggestions that the helical shaped bacterium corkscrews its way through the mucus, previous studies from our lab showed that bacteria are immobile in mucin gels in the absence of urea^11^, as well as in other gels such as gelatin^12^. Celli *et al*.^11^ showed that the elevation of pH due to urease activity also leads to a de-gelation in mucin gels^13,14^ producing a liquid environment through which bacteria can swim. Although this has not been verified in mucus, it is likely that the elevation of pH plays an important role in the ability of the bacterium to get across the mucus layer since mucin is the dominant protein responsible for the viscoelasticity of mucus.

It is also well known that *H*. *pylori* express adhesins which bind to specific antigens present both on secreted mucins in the mucus layer and on glycolipids and membrane bound mucins in the cell surface glycocalyx^15–17^. *H*. *pylori* strains vary in their proliferative response to mucins and interactions with mucin can modify *H*. *pylori* expression of adhesins genes^18,19^. This demonstrates that mucins can regulate the behavior of *H*. *pylori* beyond acting merely as a physical barrier and attachment site. *H*. *pylori* can bind to human gastric mucins via at least three modes of adhesion: via the blood group binding adhesin (BabA) and the sialic acid binding adhesin (SabA) that bind the Lewis b (Le^b^) and sialyl- Lewis a/x (SLe^x/a^) terminating glycans present on mucins, respectively, with a pH optimum close to neutral, and to charged structures with an acidic pH optimum^15,20^. The charge dependent binding mechanism at acidic pH appears common to all strains investigated so far, whereas the strains differ in expression of the SabA and BabA adhesins^16,21^. Conversely, the host also differs in expression of the corresponding structures, with the Le^b^ structure being governed by the genotype of the host whereas the presence of the SLe^x/a^ and charged structures among mucin glycans largely are governed by the inflammatory status of the host stomach^15^.

One of the dominant factors controlling bacteria motility is the viscosity of the medium, which governs the hydrodynamic interactions between the swimmer and the fluid it swims in. Early experiments using bacteria^22–26^, spirochetes^27–29^, and spiroplasma^30^ showed a complex dependence of swimming speed with increasing medium viscosity^22,31,32^. We refer to a recent review from our group^33^ for a simple explanation of the basic concepts of rheology and to a review by Yang *et al*.^34^ for the different techniques used. Briefly, rheology measures the elastic response of a solid to deformation and the viscous response of a fluid as it flows. Complex heterogeneous media such as mucus and mucin exhibit a viscoelastic response which varies with the frequency of the applied stresses and their viscosity decreases with increasing shear rate applied to the fluid^14,35^, i.e. they are shear thinning materials. The viscoelastic behavior of mucins depends on pH, ionic strength, and degree of hydration and thus is particularly relevant to the transport of bacteria through mucus. Moreover, in a heterogeneous medium whose structure is not spatially uniform, the rheological response depends on the length scale over which the medium is deformed. The local microrheological response can be probed by directly tracking the diffusive, random motion (also known as Brownian motion) of micron-size polystyrene latex particles using conventional optical or fluorescence microscopy because the average mean square displacement (MSD) of the particle as a function of time is directly related to viscosity as discussed below. Moreover, using a generalized Langevin equation it is possible to extend the analysis to viscoelastic media and obtain the frequency dependent viscous and elastic moduli characterizing the response to an oscillatory shear stress^34,36^. Previous studies of micro-particle tracking in mucin^33,37,38^ have shown the dependence of mucin viscoelasticity on concentration, pH and other factors. Furthermore, in the presence of swimming bacteria the Brownian motion of the probe particles is itself changed due to the hydrodynamic coupling of the active flagella driven motion of bacteria with the passive Brownian motion of the probe particles^39^.

The motility of *H*. *pylori* in broth, polymer solutions, and purified porcine gastric mucin (PGM) solutions has been studied by several researchers^11,40–42^. Our recent work^43,44^ explored the dependence of *H*. *pylori* motility on cell shape (helical versus rod), cell body length, helicity, variation in number of flagella, and the influence of medium viscoelasticity. However, all of those experiments were done either in culture broth or in PGM solutions, which is not the native mucin in which *H*. *pylori* lives. In this paper, we use phase contrast optical microscopy to compare the motility of *H*. *pylori* in a collection of human gastric mucin samples isolated from surface and gland mucus of healthy subjects and mucin isolated from one tumor sample. Additionally, we also examine whether the surface and gland mucins differ in their rheological properties by measuring the microrheological properties of human mucins in the absence and presence of bacteria using particle-tracking microrheology. As these mucins also varied in binding to *H*. *pylori* adhesins, we compared the motility of the wild type J99 with an adhesin deletion mutant J99Δ*babA*Δ*sabA* lacking the blood group binding adhesin (BabA) and the sialic acid binding adhesin (SabA) that bind the Le^b^ and SLe^x/a^ respectively. We examine whether motility is influenced by the location of the mucin as well as due to specific interactions between the bacterium and its native mucins. For comparison we also examined the motility of these bacteria in culture broth and PGM. We note, that to the best of our knowledge, the results presented here are the first measurements of microrheology and motility of *H*. *pylori* in human gastric mucins, as previous studies have been conducted with synthetic polymer solutions and PGM.

## Results and Discussion

### Mucin tissue location and binding to isolated mucins

**Figure 2:**
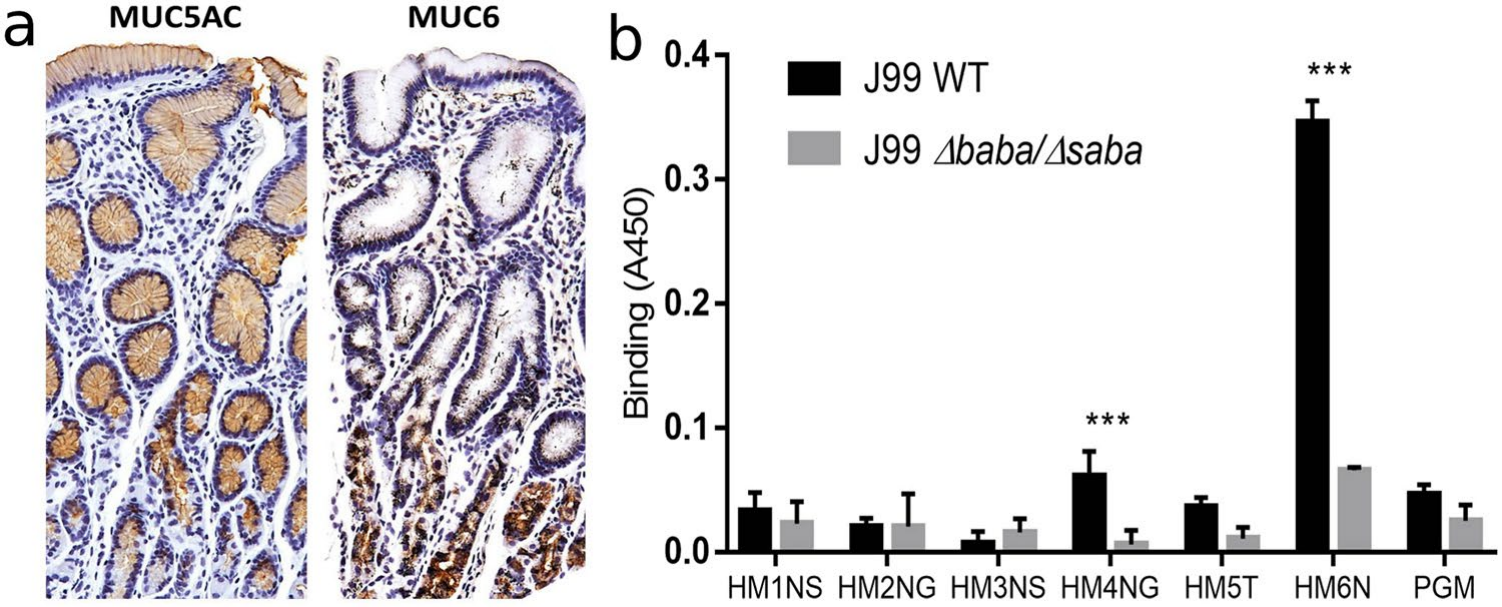
Location of mucins with antibody staining. (**a**) Left: Surface and foveolar epithelial cells stained brown with an antibody recognizing MUC5AC. Right: gland cells stained brown with an antibody against MUC6. The sections were counterstained with heamatoxylin, outlining the tissue in blue, with the dark blue representing nuclei. (**b**) Binding of J99 WT and its isogenic adhesin deletion mutant J99Δ*babA*Δ*sabA* to mucins from five patients with varying glycosylation and health status (outlined in Table 1) and to pig gastric mucin (PGM). The *** indicates p < 0.001 difference in binding between J99 WT and J99Δ*babA*Δ*sabA* to a mucin (Two way ANOVA). The results are shown after subtracting the background signal for the assay in the absence of mucins. The level of binding was relatively weak in comparison to what we previously have showed for some other human mucins^18^, although the positive control human mucin (HM6N in the graph) displayed high binding to *H*. *pylori*, demonstrating that the low binding is not due to technical issues, but due to the properties of the mucins investigated in this study.

In the gastric mucosa, MUC5AC is present in the surface and foveolar epithelium and MUC6 in the glands, and samples isolated from the surface contained mainly MUC5AC whereas mucins isolated from the glands mainly contained MUC6 (Fig. 2a and Table 1). Of the six mucins investigated in this study, Le^b^/SLe^x^ dependent binding to *H*. *pylori* strain J99 via BabA/SabA occurred to Sample HM4NG (Fig. 2b), albeit the level of binding was relatively weak compared to what we previously have demonstrated for other human mucins^18^. In the *in vivo* mucus layer, other molecules such as trefoil factors and IgA could potentially contribute to interactions between *H*. *pylori* and mucins^45^. However, the mucins studied here have been isolated using a chaotrophic agent followed by density gradient centrifugation. This procedure disassociates non-covalent associations and removes the majority of non-mucin molecules from the purified mucins (Supplementary Information Fig. S1). Together with the observation that most mucins studied here did not bind to *H*. *pylori*, this suggests that the only interaction studied here is the direct interaction between *H*. *pylori* and mucins.

**Table 1:** Summary of mucin samples: the mucin names ending in S were obtained from the surface of the stomach mucosa and the ones ending in G from the glands.

	Le^b^	Sialic acid	MUC5AC	MUC6	Health	η [cP]
HM1NS	−	−	+ +	+	Normal	1.83 ± 0.15
HM2NS	+ + +	−	+ + +	+	Normal	1.95 ± 0.30
HM3NG	−	−	+ +	+ + +	Normal	2.27 ± 0.56
HM4NG	+ + +	−	+	+	Some Inflammation	2.06 ± 0.31
HM5T	+ +	+ +	+	+ +	Tumor	1.26 ± 0.12
HM6N	+	−	+ +	+	Normal	ND
PGM^†^						9.0 ± 2.0

HM6N is from antrum and contains material from both glands and surface. Samples were selected based on being negative for the α1, 4 GlcNAc structure that has been demonstrated to have antibiotic activity^60^, to avoid this complicating factor, and therefore the gland and surface samples do not come from the same patient; “+” indicates the level of presence and “−” indicates the absence of particular structure. Data for positive control human mucin (HM6N) are included. Viscosity of mucin solutions at 15 mg/ml, **η**, given as average ± standard deviation (see text). ^**†**^The viscosity of 15 mg/ml PGM solution reported here is from Martinez *et al*.^43^. ND indicates not determined.

### Microrheology of mucins

To directly measure the microrheological properties of human mucin, we probed the diffusive, random motion of micron-size polystyrene latex particles using fluorescence microscopy. To calculate the viscosity of the medium we note that MSD at time *t* for random diffusive motion in 2-dimensions is related to the diffusion constant *D*_0_ of the particle by,

1 $$MSD(t)=4{D}_{0}t$$

Since the MSD grows linearly over time, the slope (*m*) of average MSD vs. time graph is *4D*_0_. For particles undergoing passive Brownian diffusion, the diffusion constant is related to the hydrodynamic friction coefficient ζ via the Einstein relationship which in turn is proportional to the viscosity of the medium *η* via the Stokes-Einstein relation for spherical particles of radius *a*,

2 $$D=\frac{kT}{\zeta }=\frac{kT}{6\pi \eta a}$$

Here *k* is the Boltzmann constant, *T* is the temperature in °K. Combining Eqs 1 and 2 we can calculate the viscosity from the slope *m* of the average MSD vs. time graph,

3 $$\eta =\frac{2kT}{3\pi ma}$$

**Figure 3:**
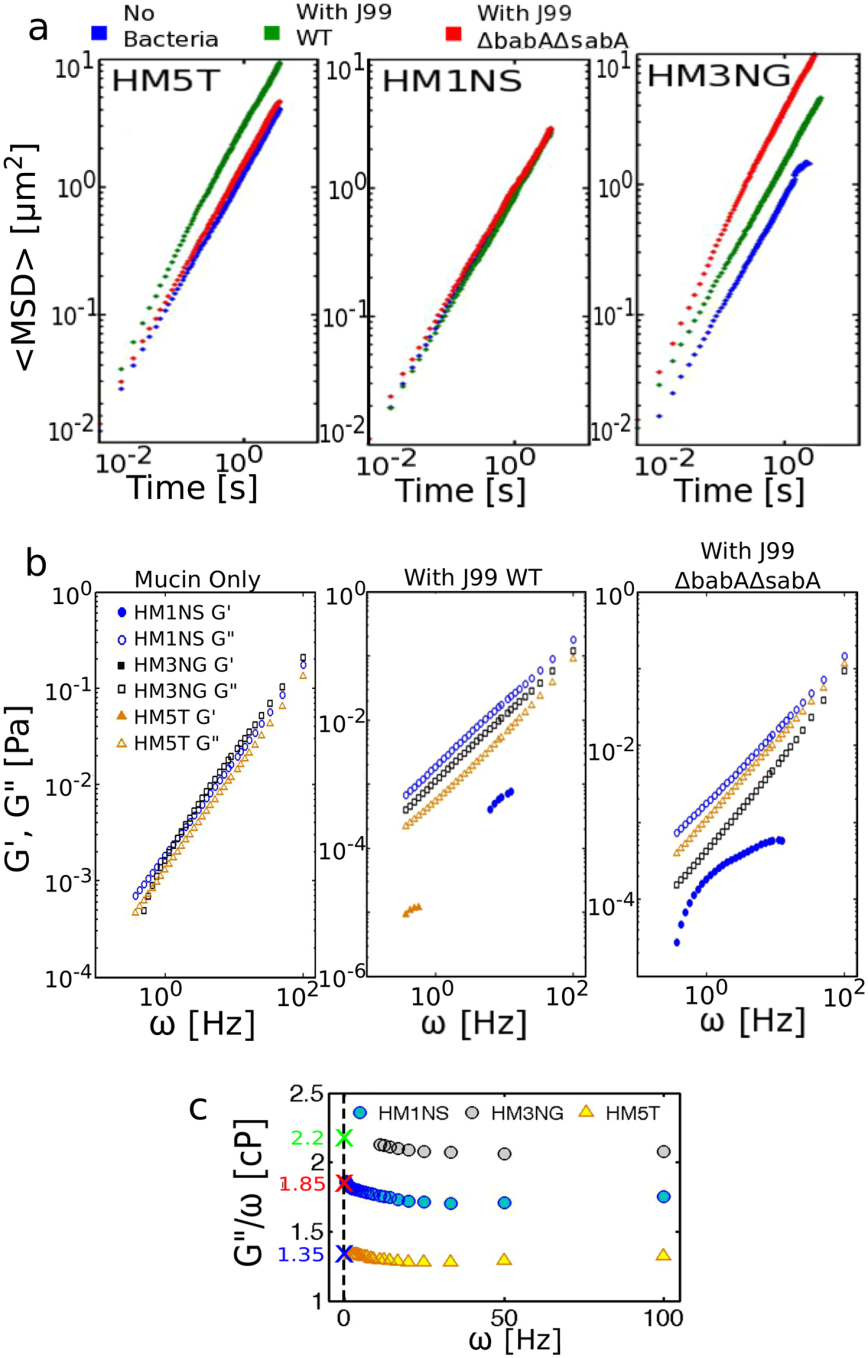
Particle tracking microrheology results from different human mucin solutions at concentration of 15 mg/ml. (**a**) Average MSD of particles in HM5T (left panel), HM1NS (middle panel), and HM3NG (right panel) in absence of bacteria (blue), in presence of J99 WT (green), and in presence of J99Δ*babA*Δ*sabA* (red). (**b**) Elastic and viscous moduli, G′(ω) (filled symbols) and G′′ (ω) (hollow symbols) of HM5T (orange), HM1NS (blue), and HM3NG (black) without bacteria (left panel), in presence of J99 WT (middle panel), and in presence of J99Δ*babA*Δ*sabA* (right panel). (**c**) Ratio, G′′(ω)/ω, of HM1NS (blue), HM3NG (black), and HM5T (orange). Colored crosses indicate the limiting values of η at zero frequency.

It is important to note that in the presence of active swimmers the passive Brownian motion of particles is enhanced^39,46^ and the Stokes Einstein equation does not apply, hence it is not possible to calculate an effective viscosity. In this case we calculate the diffusion coefficient D_+bac_ and compare to diffusion coefficient of particles in the absence of bacteria, D_0_ (Table 2). The MSD as a function of time for individual particles tracked in HM5T, HM1NS, and HM3NG mucin solutions (15 mg/ml) as well as in mucin solutions containing bacteria (J99 WT or J99Δ*babA*Δ*sabA*) are presented in the Supplementary Information Fig. S2. Figure 3a shows the average MSD of these mucin solutions on log-log plots in the absence of bacteria and in the presence of actively swimming bacteria. Table 1 gives the average viscosity values ($\eta$) obtained for the different human mucin samples using Eq. (3), along with the standard deviation calculated from analyzing individual MSD curves in the absence of bacteria. We find that the viscosity values of gland mucin solution were consistently higher than those of epithelial surface mucin (Table 1). The gland mucins consist mainly of MUC6 and the surface mucin mostly of MUC5AC. We suspect this variation in mucin composition to be a contributing factor to the viscosity difference since although MUC5AC and MUC6 both are oligomeric glycoproteins, MUC5AC could be smaller in size than MUC6^47^, which might explain the lower viscosity in surface mucin solution. We also noted that the mucin sample collected from the tumor (HM5T) has lower viscosity than other samples. Whether the decrease of viscosity of the tumor mucin solution is related to the presence of sialic acid in HM5T, which is not seen in the other mucins (see Table 1), or other differences is not clear. Truncated glycans is a common feature of cancer cells^48^, and this could be a potential cause of the decreased viscosity.

**Table 2:** Distribution of bacteria speed in different media and the influence of active bacteria on particle diffusivity.

Media	*t*_inc_	J99	N	<v_run_>	σ	v_M_	%motile	D_+bac_/D_0_	α
				[μm/s]					
BB10	45 m	WT	653	4.2	2	3.5	44.9	—	—
		Δ	796	4.3	2	3.8	63.8	—	—
PGM	45 m	WT	2363	5.1	3.6	4.1	46.2	12.6	1.3
		Δ	1944	4.5	3.2	3.7	39	7.9	1.4
HM1NS	45 m	WT	2376	3.4	1.9	3.1	4.3	0.9	1
		Δ	Immobile					1	1
	2 hr	WT	1302	4	1.7	3.6	4.5	1.2	1
HM3NG	45 m	WT	Immobile					2.1	1
		Δ	2524	9.5	6.6	7.6	75.6	5.8	1.2
	2 hr	WT	Immobile					2.3	1
		Δ	764	8.4	6.7	6	68.2	5.8	1.1
	24 h	WT	3053	5.3	3.7	4.2	52.6	2.9	1.1
		Δ	4343	3	2	2.4	19.6	2.3	1
HM5T	45 m	WT	4142	12.9	7	11.9	53.7	2.2	1.1
		Δ	Immobile					1.2	1
	2 hr	WT	5846	18.4	9.1	18.8	38.8	3.3	1.1
		Δ	Immobile					2.2	1.1
	24 h	WT	2065	8.9	6.4	6.7	64.6	1.6	1.1
		Δ	Immobile					1.2	1

The speed distribution is characterized by the mean <v_run_>, median v_M_, and its width measured by standard deviation σ. The percentage of mobile bacteria is also given. The influence of bacteria on particle diffusion is given by the ratio of particle diffusion constant in presence of bacteria (D_+bac_) to that in absence of bacteria (D_0_), and α the exponent characterizing the diffusion as normal (α = 1) or super diffusive (α > 1). Here *t*_inc_ = incubation time, N = number of trajectories averaged over.

To see the effect of enhanced particle diffusion in the presence of bacteria, we examined the average MSD of the samples containing bacteria and compared them to the average MSD of the mucin in absence of bacteria. As seen in Fig. 3a, the slope of average MSD vs *t* becomes steeper and the maximum MSD increases when motile bacteria are present indicating that the diffusivity *D*_*+bac*_ of particles increases. More specifically, in the tumor sample, HM5T, the J99 WT was much more motile than the J99Δ*babA*Δ*sabA* which were mostly immobile, and the highly active bacteria motion led to an enhanced diffusivity (Table 2). Similar results were observed in the HM3NG sample, where the J99Δ*babA*Δ*sabA* was the more motile bacterium (Fig. 3a and Table 2). However, in the surface mucin HM1NS bacteria were less motile, consequently the diffusivity did not change. We also recorded the MSD of particles diffusing in PGM, in the presence of these bacteria. We observed that the bacteria were highly motile in PGM, and in both cases the diffusion coefficient was higher than that reported for PGM in the absence of bacteria^39^ (Table 2).

The increase of diffusivity in the presence of active swimmers has been related to a change in the diffusion mechanism itself, transitioning from normal to super-diffusive motion^39,49^ characterized by a non-linear dependence of MSD on time,

4 $$MSD(\tau )=4D{\tau }^{\alpha }$$

For normal diffusion, α = 1, which returns Eq. (1). If α > 1, the particle shows enhanced displacement referred to as super-diffusion, whereas α < 1 corresponds to sub-diffusive behavior with the particles are diffusing less than normal. We fitted linear regression to log (< MSD >) vs. log(*t*) and determined the exponent α. As shown in Table 2, we observed mild super-diffusion in HM5T, HM3NG and PGM samples when motile bacteria were present. Mucin samples without bacteria or with immobile bacteria showed normal diffusion, with α ~1. In cases where highly mobile bacteria were present – such as J99 WT in HM5T or J99Δ*babA*Δ*sabA* in HM3NG – we detected super-diffusivity with α > 1 and enhanced diffusivity D_+bac_/D_0_ > 1 (Table 2). The values of α ranged from 1 to 1.2 for particles diffusing in the presence of J99 WT in the HM3NG and HM5T mucins. The α values for particles diffusing in presence of J99 WT and J99Δ*babA*Δ*sabA* in PGM were 1.3 and 1.4, respectively, higher than those in the human mucins, which is due to the higher viscoelasticity of PGM. Furthermore, these α values for the J99 strain are higher than those obtained in the presence of the LSH100 strain of *H*. *pylori* in PGM^39,43^. We did not observe α > 1, i.e. no super-diffusivity, in HM1NS, where the bacteria were much less motile than in HM5T, HM3NG, or PGM.

The particle tracking data was also analyzed to determine the viscoelasticity using established methods^34,50,51^. The frequency dependent storage and loss moduli, G′(ω) and G′′ (ω), were calculated to examine the viscoelasticity of the mucin solutions both in the absence and presence of bacteria (Fig. 3b). The results show that in the absence of motile bacteria the human mucin samples are purely viscous, i.e. they only exhibit a viscous modulus G′′. The frequency dependence of G′′(ω) in pure mucin solutions is shown in Fig. 3b (left panel). The same analysis for mucin solutions in the presence of J99 WT (middle panel) and J99Δ*babA*Δ*sabA* (right panel) are shown in Fig. 3b. We note that in presence of active bacteria motions – specifically, J99WT in HM5T solution and J99Δ*babA*Δ*sabA* in HM3NG solution – there is a noticeable decrease in the viscous modulus. The elastic modulus (G′) was detectable in HM1NS solution in presence of either J99 WT or J99Δ*babA*Δ*sabA*, while in HM5T we only found G′ in presence of J99 WT at low frequency. The gland mucin solutions HM3NG did not exhibit an elastic response in presence of either J99 WT or J99Δ*babA*Δ*sabA* bacteria. To calculate the frequency dependent viscosity η(ω) of pure mucin solutions, we plotted the ratio, G′′(ω)/ω (Fig. 3c). The limiting values of η at zero frequency (indicated by the colored crosses in Fig. 3c) are well within error bars of those obtained from the analysis of the average MSD plots (shown in Table 1).

We note that this collection of human mucins at 15 mg/ml did not undergo a sol-gel transition when their pH was decreased from neutral to ~pH4. The lack of an elastic modulus in purified human mucin solutions at pH 6 and no gelation at low pH are both in contrast to the viscoelasticity displayed by PGM solutions^14,33^, and the pH-dependent sol-gel transition seen in PGM^14,52^ forming an elastic gel at pH4 and lower. A few factors during the mucin preparation might have led to this outcome. The crude mucus samples were homogenized in the case of human mucin preparation while this was not done in the PGM preparation. Moreover, during PGM preparation a cut-off filter is used to selectively retain larger molecules of the mucins, which would promote gelation. Furthermore, although both mucin isolation methods involve density gradient centrifugation using CsCl, the human mucin preparation uses the chaotrophic agent guanidinium HCl (GuHCl), which disassociate non-mucin molecules from the mucins before the CsCl density gradient centrifugation. As discussed in Methods the samples were repeatedly dialyzed to remove excess ions. Waigh *et al*.^53^ have shown that mucin treated with GuHCl does not gel. Lastly, the human mucin samples were kept as solutions, whereas PGM was lyophilized. It is also possible that human mucin would gel at higher mucin concentration than 15 mg/ml. We were not able to test this as it needed a larger quantity of sample.

### Motility of J99 WT and its adhesin deletion mutant in different media

To investigate how the bacterial motility was affected by the location of the mucin sample, and how the bacterial motility and mucin solution viscosity were correlated, we tracked the motion of bacteria using time-resolved phase contrast microscopy^43^. The motility studies were conducted in human mucin solutions (15 mg/ml) as well as Brucella culture broth (BB10) and PGM (15 mg/ml) to compare with previous motility studies of other strains of *H*. *pylori*^43^. We found that both J99 WT and J99Δ*babA*Δ*sabA* were motile in broth and PGM, implying that the mutation does not alter motility in these two media. Sample movies are presented in Supplementary Information (Movie S3). However, the bacteria were not always motile in the human mucins even though bacteria taken from the same liquid culture just prior to assaying for motility were swimming well in the reference broth. Moreover, in contrast to experiments in broth or PGM where J99 WT and J99Δ*babA*Δ*sabA* always behaved in a similar manner, the individual human mucins differed both in their overall effect on swimming and in that some of them affected the J99 WT and J99Δ*babA*Δ*sabA* differently. As indicated in Table 2, J99 WT was motile in HM5T, while in HM3NG they exhibited an incubation time dependent motility. In this case motile bacteria were only observed at 24 hours, not at 45 minutes or 2 hours implying that they became motile sometime after 2 hours. In HM1NS, J99 WT was immotile at pH6, however a few swam when pH 4 buffer was added to HM1NS, and these low pH data are included here. The mutant J99Δ*babA*Δ*sabA* was motile in HM3NG but none swam in HM5T and very few swam in HM1NS (which we were not able to record). To confirm that these observations were not an experimental artifact, all the motility experiments were done at least twice and the diversity of motile behavior was observed. These surprising differences between the motility in human mucins as compared to PGM or BB10 could possibly be related to how the human mucins affect *H*. *pylori* gene expression in a BabA dependent manner^18^.

### Speed distributions, reversals, and reorientation angles

In this section we present a detailed analysis of the trajectories of J99 WT in HM1NS, HM3NG and HM5T, and J99Δ*babA*Δ*sabA* in HM3NG, as well as compare to their swimming in broth and PGM. Visual inspections of the movies showed that all bacteria displayed widely varying swimming speeds as can be seen in a typical movie (See Supplementary Information Movie S3). From the movies the bacteria are tracked and the trajectories analyzed. The bacteria tracks show overall similarity with the results reported for LSH100 and PMSS1 *H*. *pylori* strains^12,43^, exhibiting straight runs and re-orientation events. Here we define *reorientation* when bacteria change the direction that they are traveling. When the reorientation angle, θ_re_, is greater than 110° it is considered *a reversal*. We analyzed the bacteria tracks using the same methods as described in Martinez *et al*.^43^ to obtain the distributions of instantaneous swimming speeds, v_ins_ (the speed between two adjacent points on a trajectory) calculated at each time point along each trajectory, as well as the run speed, v_run_ (the average speed over the linear path between reorientation events) and the re-orientation angles.

**Figure 4:**
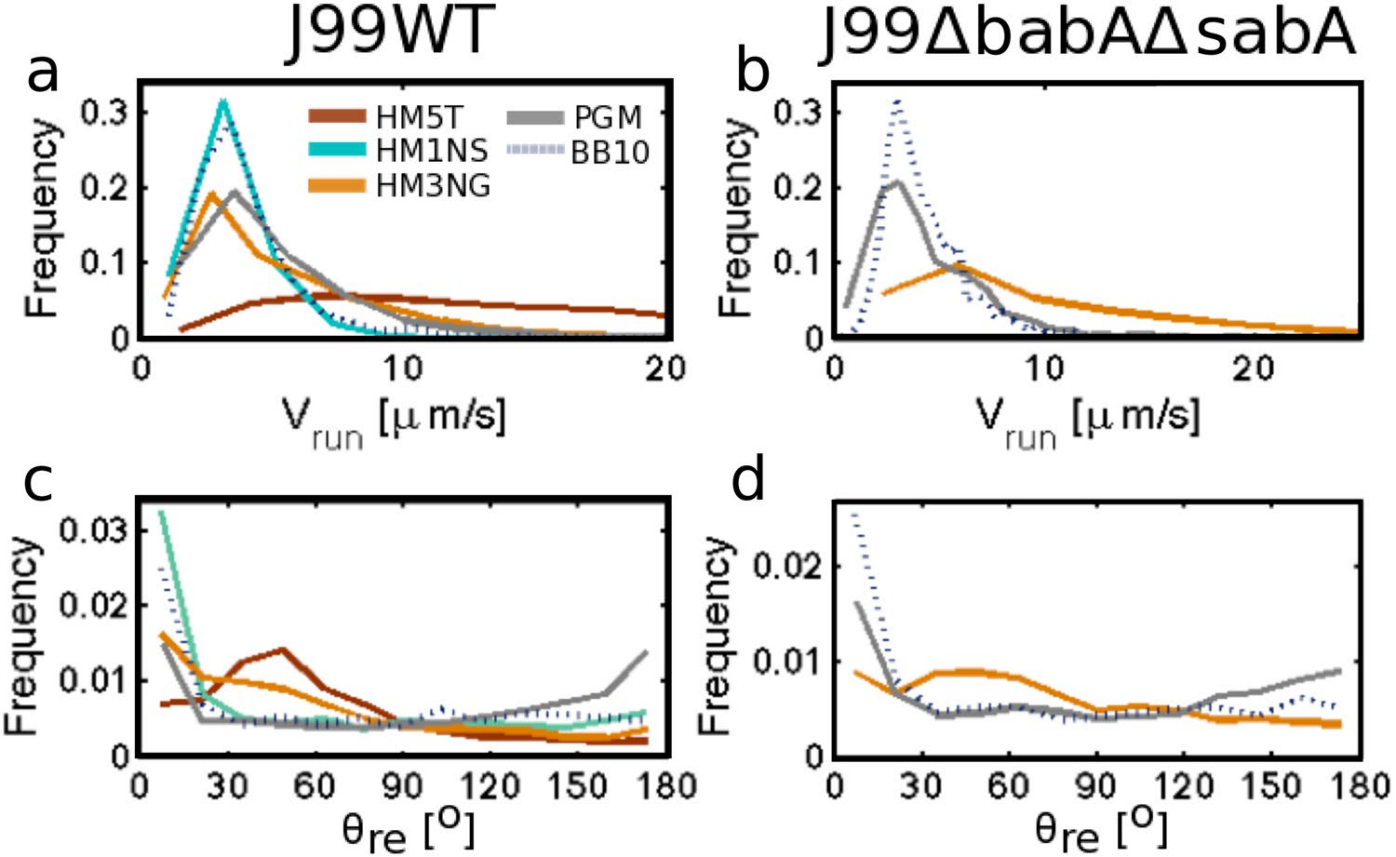
Analysis of trajectories of J99 WT and J99Δ*babA*Δ*sabA* swimming in HM1NS, HM3NG, HM5T human mucin solutions (15 mg/ml), and in PGM (15 mg/ml), and BB10. Smooth histograms summarizing the run speed (v_run_) distributions of J99 WT (**a**) and J99Δ*babA*Δ*sabA* (**b**) and the reorientation angle (θ_re_) distributions of J99 WT (**c**) and J99Δ*babA*Δ*sabA* (**d**).

Figure 4a,b show the distributions of v_run_. The instantaneous speed (v_ins_) distributions are presented in Supplementary Information Fig. S5. Both of these swimming speed distributions are very broad, reflecting the morphological heterogeneity in bacteria size, shape, and flagella number as well as temporal variation of speed, in agreement with observations for other strains of *H*. *pylori*^43^. The standard deviation of the speed distribution, **σ**, calculated over the ensemble of all bacteria tracks, can be taken as a good measure for the width of the distribution. The mean, median and standard deviation of the distribution of run speeds are presented in Table 2 and the instantaneous speed distribution in Supplementary Information Table S4. The distributions were compared using Kolmogorov-Smirnov test; when P-values are <0.05, the differences between distributions are considered statistically significant. The run speed distributions of both J99 WT and J99Δ*babA*Δ*sabA* in broth and in PGM are comparable (P = 0.5 in PGM and P = 0.49 in BB10) (see Fig. 4a,b). Figure 4a shows that the run speed distribution of J99 WT in HM1NS and broth are very similar, whereas in HM3NG and PGM there is a larger proportion of faster swimmers compared to broth. On the other hand J99 WT in HM5T differs from all other mucin solutions by exhibiting a more or less uniform distribution of run speeds. As mentioned earlier, J99Δ*babA*Δ*sabA* only swam in HM3NG out of the human mucin samples and showed a broader run speed distribution with a larger fraction of faster swimmers than in broth or PGM. Comparing the instantaneous speed distribution (Fig. S5) to the run speed distribution of J99 WT and J99Δ*babA*Δ*sabA* in BB10, PGM, and human mucins we found no differences between the two distributions in all cases, with K-S tests of all giving P ≥ 0.05 (Supplementary Information Table S6).

To investigate in greater detail the reorientation and reversals, we analyzed the bacteria tracks in human mucins and compared with reorientations and reversals in BB10 and in PGM. Figure 4c,d show distributions of the reorientation angle (θ_re_) of J99 WT and J99Δ*babA*Δ*sabA* in various media. As shown in Fig. 4c, the WT bacteria showed most reversals in PGM as compared to broth and human mucins, in agreement with previous findings on other strains in PGM^43^. We also noticed a peak at intermediate angles ~50° indicating a preferred reorientation angle which is most pronounced in HM5T, considerably decreased in HM3NG, and almost absent in HM1NS. A slight peak was also observed for J99Δ*babA*Δ*sabA* swimming in HM3NG (Fig. 4d). The preferred reorientation peak in HM5T is also more pronounced than the weak maximum observed for LSH100 and PMSS1^12^.

**Figure 5:**
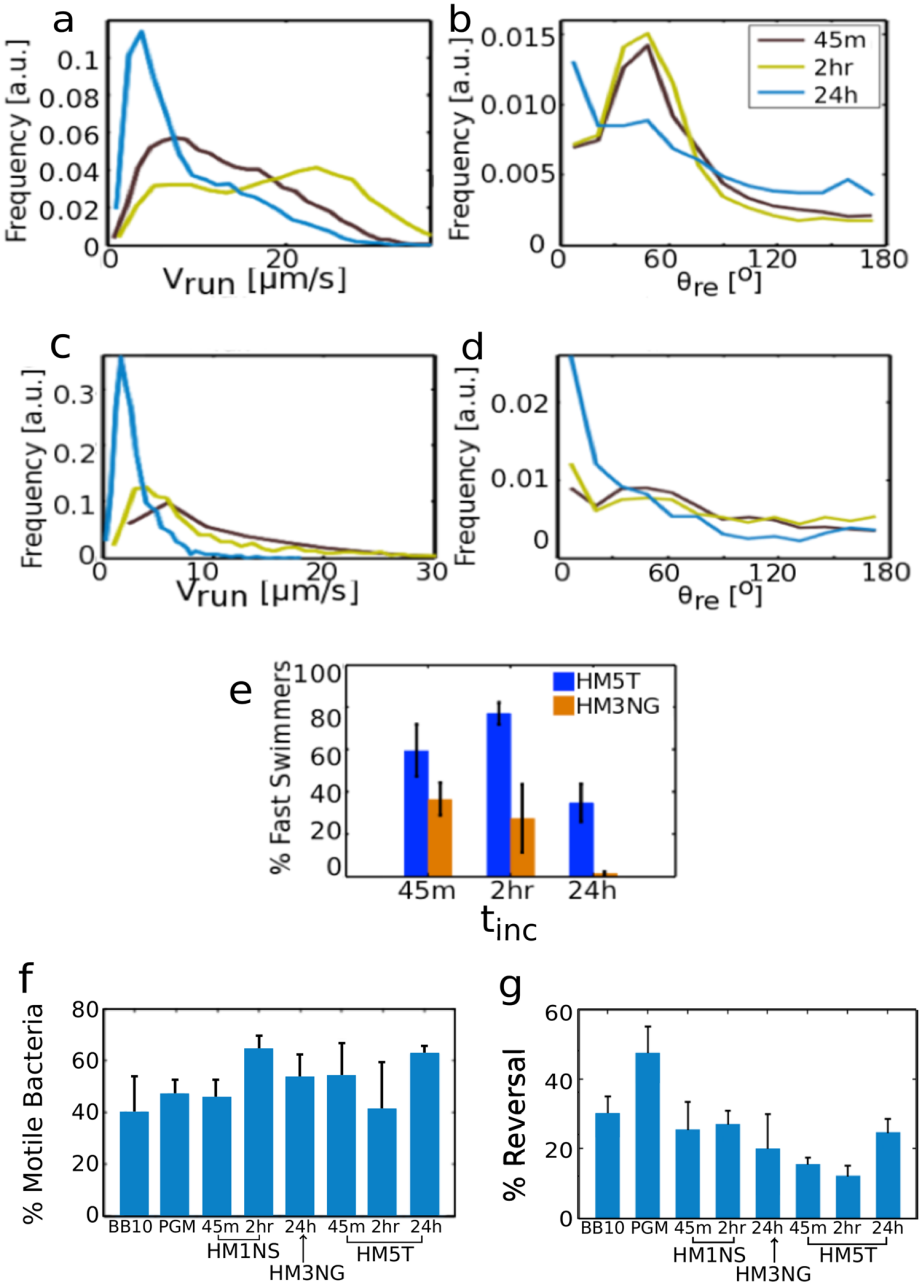
Time dependence of motility of J99 in different media. Smooth histograms summarizing run speed distributions and reorientation angle distributions of J99 WT in HM5T mucin solution (**a**,**b**) and J99Δ*babA*Δ*sabA* in HM3NG mucin solution (**c**,**d**), over 45 minutes, 2 hours, and 24 hours incubation period, *t*_inc_. Bar graphs showing time evolution of percent fast swimmers, J99 WT (blue) and J99Δ*babA*Δ*sabA* (orange) in HM5T and HM3NG (**e**); percent motile J99 WT in broth, PGM, and human mucin solutions (**f**); percent reversal of J99 WT in broth, PGM, and human mucin solutions (**g**).

To address whether the motility is altered over a period of 24 hours, we measured the motility of J99 WT in HM5T mucin and J99Δ*babA*Δ*sabA* in HM3NG mucin at three different times and present these results in Fig. 5. We note that J99 WT in HM5T gained a swimming advantage from 45 minutes to 2 hours (see Fig. 5a), although θ_re_ distribution was unchanged (Fig. 5b). At 24 hours the bacteria trajectories change, showing a marked reduction in the fraction of faster swimming speeds, and the frequency of preferred reorientations also decreases with a slight increase in reversals. Similar observations regarding the decrease of fast swimmers at 24 hours and the disappearance of preferred orientation angles were made with J99Δ*babA*Δ*sabA* in HM3NG (Fig. 5c,d). Overall, there was a larger fraction of faster swimmers (bacteria traveling at speeds ≥ 10 μm/s) of WT in HM5T than of J99Δ*babA*Δ*sabA* in HM3NG (Fig. 5e). We also calculated the percentage of mobile bacteria, defined as bacteria with MSD > 0.3 μm (Fig. 5f). As can be seen in Fig. 5f, there is no clear systematic trend in percentage of mobile swimmers over time (ranging between 40 to 60%), perhaps because the bacteria are dividing with increasing time and/or changing their motility because, for instance, the flagella could fall off or the motor could get impaired, or amount of nutrients decreased. Figure 5g shows that the percent of reversals calculated by counting over all trajectories for J99 WT is less in human mucins as compared to PGM.

**Figure 6:**
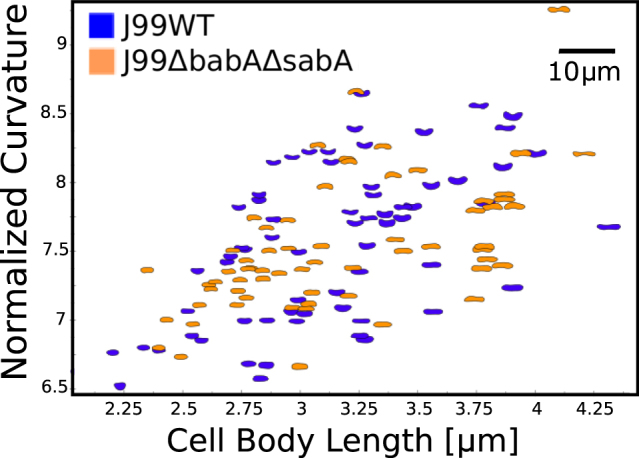
J99 WT and J99Δ*babA*Δ*sabA* display similar cell morphology. Normalized curvature versus cell length of individual bacterium imaged using phase contrast microscopy of J99 WT (blue, n = 66) and J99Δ*babA*Δ*sabA* (orange, n = 63).

To characterize the bacteria morphology we used CellTool^54^ to determine the contours of about 60 bacteria taken from single frame images at 40X from many different videos. Figure 6 shows the contours of all bacteria plotted as normalized curvature *versus* cell body length calculated as in ref.^43^. To compute normalized curvature, the average of curvature measured at each point along the cell contour excluding the poles is calculated then multiplied by the contour length, which provides a dimensionless measure of curvature by comparing the curvature of the actual cell contour with that of a circle whose circumference is equal to the contour length. Both J99 WT and J99Δ*babA*Δ*sabA* show a broad heterogeneous distribution of lengths and curvatures as reflected in standard deviation (σ) for the length distribution σ = 0.47 μm for J99 WT and 0.48 μm for J99Δ*babA*Δ*sabA*, and curvature distribution σ = 2.0 for J99 WT and 2.2 for J99Δ*babA*Δ*sabA*. The two bacteria have almost identical average body lengths of 3.30 μm and 3.34 μm, and only slightly different curvatures of 7.56 and 7.43 for J99 WT and J99Δ*babA*Δ*sabA*, respectively. A comparison of our measurements with those reported earlier^43^ on LSH100, PMSS1 and B128 shows that J99 is similar in length to PMSS1, but more curved than all three previously studied strains.

## Conclusion

To the best of our knowledge, we report the first results of microrheology and motility of *H*. *pylori* in human mucin solutions by tracking particles and bacteria. The microrheology measurements reveal that the viscosity of mucin solutions is dependent on the location in the mucus layer from where the samples were collected, with gland mucin being more viscous than the epithelial surface mucin and the tumor mucin having the lowest viscosity. In contrast to PGM, we observed no elastic response in any of the human mucin solutions in the absence of bacteria. The presence of bacteria enhances the particle diffusivity, and produces an elastic response in some cases. The particle diffusivity increased the most for the tumor sample (3.3 times), was intermediate for the gland mucin (2.9 times), and least for the surface mucin (1.2 times). We observed mild super-diffusivity of particles in samples where bacteria were highly motile in agreement with recent observations that the active motion of bacteria produces convective effects that enhance passive Brownian motion of particles^46^. The detection of an elastic response in HM1NS solution in presence of J99 WT or J99Δ*babA*Δ*sabA*, and in HM5T in presence of J99 WT at low frequency is further suggestive of the influence of bacteria motion on medium rheology.

We observed that while both J99 WT and J99Δ*babA*Δ*sabA* were motile in PGM solutions and broth, their ability to swim in human mucins varied considerably with bacteria being immotile in some of the human mucin solutions. The motility of J99 WT and adhesin mutant were impacted differently by different mucins. In those cases where bacteria swam, we found significant differences in motility in human mucin solutions as compared to broth and PGM. The speed distributions of both WT and adhesion deletion mutant of J99 in human mucin solutions are broader and have a larger fraction of faster swimmers and less reversals in comparison to PGM or broth.

While our work provides valuable data on human mucin rheology and motility of *H*. *pylori* it also raises unanswered questions worthy of further investigation. First, we could not correlate differences in motility systematically to differences in adhesion. The bacteria studied here had relatively weak BabA dependent binding and this may be another factor contributing to our observation of no systematic effect of adhesion on motility. We also cannot explain why J99Δ*babA*Δ*sabA* were immotile in both HM5T and HM1NS while WT J99 was immotile in HM3NG for the first 2 hours but became motile at 24 hours. This immotility is not due to a general toxic effect, since either the J99 WT or J99Δ*babA*Δ*sabA* swam in all tested mucins and both types of bacteria swam well in broth and PGM. We believe that their immotility in some of the human mucins probably reflects influence of other non-defined factors present in human mucin samples. For example, could these differences be related to that human mucins differ in how they affect *H*. *pylori* gene expression in a BabA dependent manner^18^? In the same vein, we cannot explain why an elastic response was detected in the presence of motile bacteria in HM1NS and HM5T solutions but not in the presence of HM3NG.

Although we have only examined a single tumor sample, our results suggest that the tumor mucin investigated here may be altered in comparison to non-tumor surface and gland mucins leading to a decreased viscosity which enhances the motility of *H*. *pylori*. Overall, these results suggest that mucins from different individuals, tissue locations and disease status differ in how they affect *H*. *pylori* motility. Further studies investigating these issues in healthy and tumor mucin samples to examine the interplay between mucin viscosity and *H*. *pylori* motility are clearly warranted.

## Methods

### Culturing conditions

*H*. *pylori* strain J99 WT and its isogenic knockout mutant J99Δ*babA*Δ*sabA* lacking blood group antigen binding adhesins (BabA), which binds primarily to Le^b^ and related mucin structures, and sialic acid binding adhesins (SabA), which bind mainly to sialyl-Lewis x (SLe^x^) and sialyl-Lewis a (SLe^a^) were kindly provided by Prof. Thomas Borén, Umeå University, Sweden^21^. Both J99 WT and J99Δ*babA*Δ*sabA* were cultured on Brucella agar (Brucella Medium Base, Oxoid, Basingstoke, Hampshire, England) supplemented with 10% sheep blood, 1% IsoVitox (Oxoid), 4 mg/L amphotericin B, 10 mg/L vancomycin and 5 mg/L trimethoprim, then inoculated in liquid media containing 10% fetal bovine serum and 90% Brucella broth. Plates were maintained at 37 °C under microaerobic conditions in a tri-gas environment using BD GasPak systems. Cultures were incubated for 45–72 hours on sheep blood plates and for 12–18 hours in liquid cultures on the shaker.

### Human mucin preparation and binding affinity studies

#### Mucin isolation and characterization

Mucins were isolated from five gastric specimens. Four of these were macroscopically normal and obtained after written informed consent (Ersta Diaktioni, Sweden) in conjunction with obesity surgery and the regional ethics board (Regionala etikprövningsnämnden I Göteborg, Dnr 753-14) has approved the use of the human samples for investigating interactions between *H. pylori* and human mucins. The fifth specimen was from a tumor sample from our well characterized mucin library, collected in 1983 at the IMIM-Hospital del Mar, Barcelona, Spain (before the hospital had an ethics committee).

Three of the samples were deemed normal based on histology, one of the samples showed signs of inflammation and one sample was obtained from a gastric adenocarcinoma tumor located in the antrum. The health condition of the samples is summarized in Table 1. Mucins were solubilized in 6 M GuHCl, purified using isopycnic CsCl density gradient centrifugation and concentration as well as mucin species and glycosylation was determined using microtitre based assays, as previously described^19^. The sialic acid content reported in Table 1 is a combination score for several assays: sample HM1-4 were determined negative for sialic acid by absence of alcian blue stain by histology and absence of signal in an Enzyme linked immunosorbent assay (ELISA) for sialyl-Le^a^. HM5T was determined sialic acid containing by positive signal for sialyl-Le^a^ by ELISA, and sialic acid containing structures were also confirmed using mass spectrometry^18,55^. Similarly, HM6N was determined negative for sialic acid by absence of signal for sialyl-Le^a^ by ELISA, and absence of sialic acid containing structures by mass spectrometry^18,55^. Isolated mucins were dialyzed against 3 changes of 2 M NaCl followed by 3 changes of PBS to ensure efficient removal of harmful ions potentially remaining from the isolation procedure. All human mucin solutions were diluted to 15 mg/ml for microrheology and motility studies.

#### Bacterial binding assay

Mucin samples were diluted to 6 µg/ml in 4 M GuHCl and coated on 96-well polysorp plates overnight at 4 °C. *H*. *pylori* were grown on Brucella plates for 72 h and harvested in PBS. The bacteria were centrifuged at 2500 × *g* for 3 min and then resuspended in Blocking Reagent for ELISA (Roche). The plates were washed three times with washing buffer (PBS containing 0.05% Tween-20) and the wells were blocked for 1 hour with blocking buffer. After discarding the blocking buffer, bacteria with an OD_600_ of 0.1 were diluted 1:10 in blocking buffer and added to the plates, which then were incubated in a bacterial shaker at 100 rpm, 37 °C for 2 hours. Then the plates were washed three times with PBS containing 0.05% Tween-20, which was repeated between every subsequent incubation step. The plates were incubated with rabbit anti-*H*. *pylori* serum diluted 1:1000 in blocking buffer for 1 hour at RT, that was followed by incubation with HRP-conjugated donkey anti-rabbit IgG diluted 1:10,000 in blocking buffer for 1 hour at RT. For detection, 3,3′,5,5′-Tetramethylbenzidine (TMB) substrate was added and color development was monitored. The reaction was stopped with 0.5 M H_2_SO_4_. The absorbance was measured in a microplate reader at 450 nm.

#### Histological methods

Inflammatory status of the gastric specimen was determined on paraffin embedded hematoxylin and eosin stained sections. Tissue sections were stained for MUC6 (LUM6-3 antibody) and MUC5AC (45M1 antibody) as previously described^56^.

### PGM preparation

PGM was collected from pig stomach epithelium and purified with Sepharose CL-2B column chromatography, followed by density gradient ultracentrifugation, described in Celli *et al*.^11^ and more fully in the original reference Gong *et al*.^57^. 0.75 mg of lyophilized PGM powder was dissolved in 40 μl of sterile water, the solution reaches the final 15 mg ml^−1^ concentration after 10% bacteria and 10% pH buffer (0.1 M phosphate succinate) were added. PGM was allowed to hydrate for 48 hours in 4 °C before use.

### Microrheology measurements of human mucin in the absence and presence of *H. pylori*

Monodisperse fluorescent particles of 1.001 μm diameter (Polysciences) were added to each mucin sample (15 mg/ml) to obtain 0.054% of final particle concentration by volume for all experiments. For measurements in the absence of bacteria, mucin samples were incubated at 37 °C with fluorescent particles and 10% pH buffer (0.1 M phosphate succinate, pH2, 4, and 6) by volume for 45 minutes. For measurements in the presence of bacteria, mucin samples were first incubated at 37 °C with 10% pH buffer for 45 minutes. Bacteria were cultured in liquid broth (BB10) to an O.D._600_ of 0.55–0.85 then added to each sample to reach a 10% bacteria mixture by volume. The bacteria mixture was incubated at 37 °C under microaerobic conditions and constant agitation for 45 min, 2 hours, and 24 hours. After the incubation period, fluorescent particles were added to the samples prior to measurements. A 10 μl volume of each solution was pipetted onto a standard glass microscope slide with a spacer (Secure Seal Imaging Spacer, 9 mm in diameter, 0.12 mm in depth) and sealed with a coverslip. The movies were acquired immediately at room temperature (~21 °C) with an Olympus I71 inverted optical microscope equipped with a 40x phase contrast lens (0.65 NA) and an Andor Zyla 5.5 sCMOS camera at 100 fps and 6.5 μm per pixel size. Fluorescent particles were excited using Olympus BH2 Mercury arc source. Imaging focus was optimized at the center Z positions of each sample to minimize edge effects. Three-second videos were obtained from several different x-, y- positions with optimal z-position throughout each sample. The Brownian motion of the particles was tracked in MATLAB [v7.12.0] using PolyParticleTracker routine which locates the center of intensity of each tracked object with a polynomial Gaussian fit^58^. Mean square distance and the viscosity of each sample were calculated based on the tracker position output. Frequency dependent elastic and viscous moduli (G′ and G′′) were calculated based on previously described methods^50,51^ using the MATLAB programs provided on http://www.physics.emory.edu/~weeks/idl/.

### Bacteria motility and swimming trajectory in BB10, PGM, and human mucins

The human mucins, PGM, and reference BB10 sample were incubated at 37 °C with 10% (by volume) phosphate buffer for 45 minutes. The values of pH we explored were pH 6 and pH 4. Bacteria were cultured in liquid broth (BB10) to an O.D._600_ of 0.6–0.85 then added to each mucin solution (15 mg/ml) and reference BB10 sample to produce a 10% bacteria mixture by volume. The bacteria mixture was incubated at 37 °C under microaerobic conditions and constant agitation for 45 minutes, 2 hours, and 24 hours. After each of these incubation times, a 10-μl volume of each sample was applied to glass microscope slides with secure seal spacers and sealed with coverslips. The samples were imaged immediately at room temperature using an Olympus IX70 inverted phase contrast microscope equipped with a 40x objective lens (0.65 NA), a halogen light source, and an Andor Zyla 5.5 sCMOS camera at 33 fps and 6.5 μm pixel size. Nine-second videos of bacteria swimming in mid-plane between the coverslip and microscope glass slide were acquired using Micro-Manager open source acquisition software. Six to ten videos were taken for each sample at each of the three incubation time points. All measurements were repeated at least twice. Bacteria trajectories were tracked using PolyParticleTracker MATLAB routine. Swimming speed distributions, reorientations, and number of reversals were calculated from the tracker output using the methods of Martinez *et al*.^43^. The standard deviation of speed distribution, σ, was calculated over the ensemble of all bacteria tracks, and can be taken as a good measure for the width of the distribution. Additionally, we note that time is measured to an accuracy of $\frac{1}{frame\,rate}\,$= 0.03 s and distances are measured to an accuracy of $\sqrt{2\frac{pixel\,size}{magnification}}$ = 0.23 μm so accuracy in calculating a speed of 10 μm/s is about 5%, which is considerably smaller than σ.

### Morphology analysis: J99 WT and J99Δ*babA*Δ*sabA*

Single frame images of bacteria were manually selected using ImageJ from phase contrast movies at 40x collected for motility analysis. CellTool^54^ software was used to obtain cell length and side curvature for J99 WT (n = 66) and J99Δ*babA*Δ*sabA*, (n = 63) as described in Sycuro *et al*.^59^. The average values of length, side curvature and standard deviation (σ) of distributions of these morphological parameters were calculated.

All studies reported here on *H*. *pylori* in purified human and porcine gastric mucin were carried out under Boston University’s IBC (institutional Biosafety Committee) approval number 17–1968 (P.I. R. Bansil).

## Electronic supplementary material

Supplementary Information.pdf

Supplementary Information Movie S3. A: J99 WT in BB10

Supplementary Information Movie S3. B: J99ΔbabAΔsabA in BB10

Supplementary Information Movie S3. C: J99 WT in PGM

Supplementary Information Movie S3. D: J99ΔbabAΔsabA in PGM

Supplementary Information Movie S3. E: J99 WT in HM1NS with pH4 buffer added

Supplementary Information Movie S3. F: J99 WT in HM3NG after 24 hours of incubation

Supplementary Information Movie S3. G: J99 WT in HM5T

Supplementary Information Movie S3. H: J99ΔbabAΔsabA in HM3NG
