# Supplementary material for: Influence of the viscosity of healthy and diseased human mucins on the motility of Helicobacter pylori
Source: Sci Rep. 2018 Jun 26;8:9710. doi: 10.1038/s41598-018-27732-3 (PMC6018794; doi:10.1038/s41598-018-27732-3)
Supplement: Supplementary file 1 — Supplementary Information.pdf [file 41598_2018_27732_MOESM1_ESM.pdf]

Influence of viscosity of healthy and diseased human mucins on motility of *Helicobacter pylori*

Clover Su, Médea Padra, Maira Alves Constantino, Sinan Sharba, Anders Thorell, Sara K. Linden, Rama Bansil*

Supplementary Information

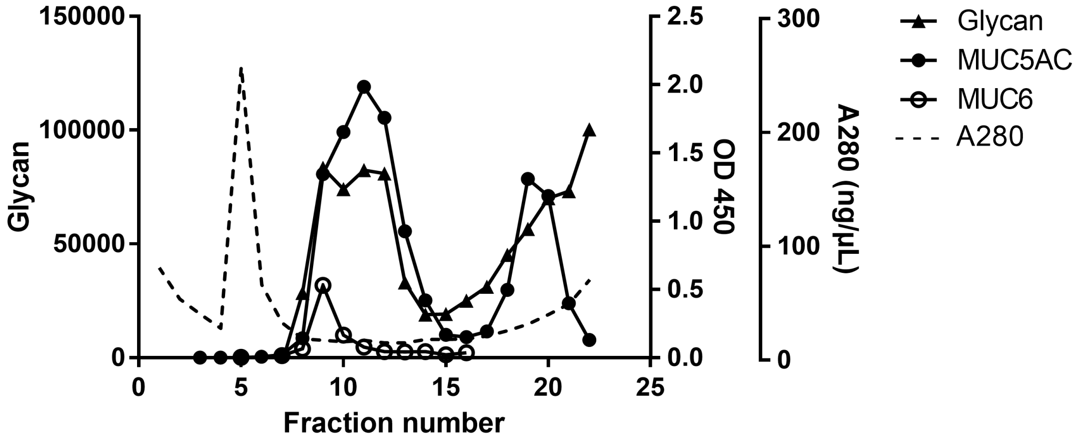

Supplementary Information Figure S1. Mucin isolation and characterization. Mucins were isolated using a chaotrophic agent (GuHCl) followed by density gradient centrifugation. The fractions were emptied from the bottom of the tube, and the density is thus highest in the fractions with low numbers. The isolation procedure disassociates non-covalent associations and removes DNA and the majority of non-mucin molecules from the purified mucins, as can be seen by the absorbance at 280 nm: there is a peak containing DNA at fraction five, which decrease to baseline before the fractions containing the mucins start (fraction 9-13), and the A280 signal starts to increase again after fraction 15. The increase in A280 containing material at the low density fractions represent non-mucin proteins. The mucin containing fractions were pooled based on the glycan curve into one sample for each patient. The example shown in the graph is the human normal sample 1 and fraction 9-13 was pooled for further studies on the mucins.

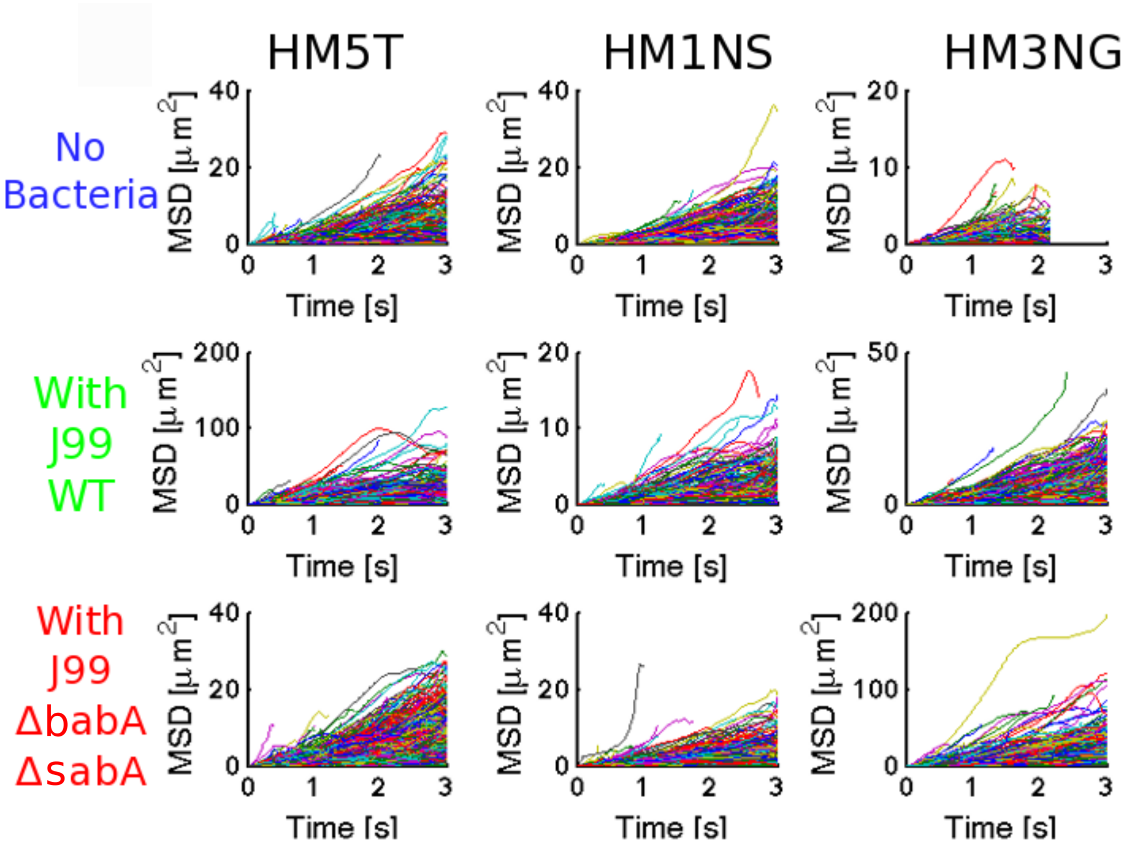

Supplementary Information Figure S2. Microrheology results from tracking particles in different human mucins. Individual MSD of particles in tumor mucin sample (HM5T), surface mucin (HM1NS), and the gland mucin (HM3NG) with or without J99 WT or J99Δ*babA*Δ*sabA*.

Supplementary Information Movie S3. A collection of sample videos showing the motility of J99 in brucella broth, PGM, and human mucins.

A: J99 WT in BB10

B: J99Δ*babA*Δ*sabA* in BB10

C: J99 WT in PGM

D: J99Δ*babA*Δ*sabA* in PGM

E: J99 WT in HM1NS with pH4 buffer added

F: J99 WT in HM3NG after 24 hours of incubation

G: J99 WT in HM5T

H: J99Δ*babA*Δ*sabA* in HM3NG

Media	T_{inc}	J99	V_{ins} [µm/s]	
			<V_{ins}>	Median
**BB10**	45m	WT	7.7	4.8
		Δ	4.8	4.5
**PGM**	45m	WT	6.1	4.8
		Δ	6.3	4.3
**HM1NS**	45m	WT	3.2	3.17
		Δ	Immobile	
	2hr	WT	4.7	4.24
**HM3NG**	45m	WT	immobile	
		Δ	12.8	11.3
	2hr	WT	immobile	
		Δ	12.7	9.8
	24h	WT	7.8	6.4
		Δ	3.7	3.1
**HM5T**	45m	WT	17.8	17.7
		Δ	immobile	
	2hr	WT	24.9	25.7
		Δ	immobile	
	24h	WT	13.7	13.2
		Δ	immobile	

Supplementary Information Table S4. Summary of the mean and median values of J99 WT and J99Δ*babA*Δ*sabA* instantaneous speeds in human mucins, PGM, and BB10 after different incubation times. T_{inc} = incubation time.

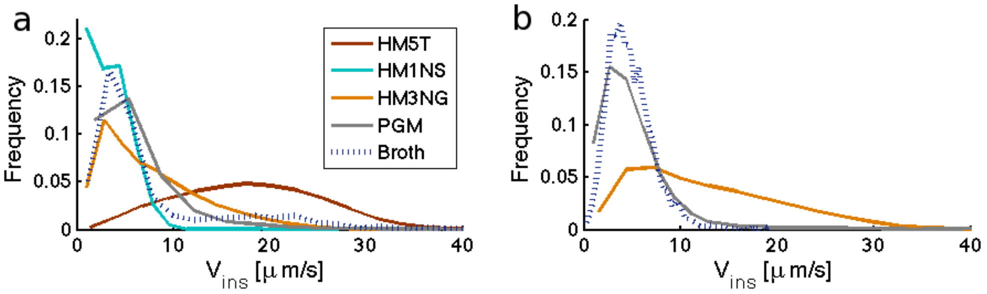

Supplementary Information Figure S5. Analysis of trajectories of J99 WT and J99Δ*babA*Δ*sabA* swimming in HM1NS, HM3NG, HM5T mucins. PGM, and BB10. Smooth histograms summarizing instantaneous speed (v_{ins}) distributions of J99 WT (a) and J99Δ*babA*Δ*sabA* (b).

K-S test: P-values

Solutions	Strain	t_{inc}	V_{runs}	V_{ins}
BB10	WT	45m	0.50	
	Δ		0.77	
PGM	WT		0.97	
	Δ		0.5	
HM1NS	WT	45m	0.5	
		2hr	0.28	
HM3NG	WT	24h	0.77	
	Δ	45m	0.97	
		2hr	0.97	
		24h	0.77	
HM5T	WT	45m	0.77	
		2hr	0.97	
		24h	0.5	

Supplementary Information Table S6. Kolmogorov-Smirnov statistical analysis comparing run speed and instantaneous speed distributions of J99 WT and J99Δ*babA*Δ*sabA* at different incubation time (t_{inc}) in HM1NS, HM3NG, HM5T human mucins, PGM, and BB10. When P-values are ≥ 0.05, the differences between distributions are insignificant.
